# Impact of pre-existing mental health diagnoses on development of post-COVID and related symptoms: a claims data-based cohort study

**DOI:** 10.1038/s41598-024-52656-6

**Published:** 2024-01-29

**Authors:** Anna Greißel, Antonius Schneider, Ewan Donnachie, Roman Gerlach, Martin Tauscher, Alexander Hapfelmeier

**Affiliations:** 1 Institute of General Practice and Health Services Research, Department of Clinical Medicine, School of Medicine and Health, Technical University of Munich, Orleansstraße 47, 81667 Munich, Germany; 2 Bavarian Association of Statutory Health Insurance Physicians, Munich, Germany; 3 Institute of AI and Informatics in Medicine, Department of Clinical Medicine, School of Medicine and Health, Technical University of Munich, Munich, Germany

**Keywords:** Risk factors, Comorbidities, Fatigue, Infectious diseases, Psychiatric disorders, Health services

## Abstract

This study aimed to examine the association of prior mental health diagnoses with the onset of Post-COVID-19 condition (PCC). We conducted a retrospective comparative cohort study and secondary analysis of routinely collected claims data from participants in statutory health insurance in Bavaria, Germany, from January 2015 to June 2022. Study participants were 619,560 patients with confirmed COVID-19, 42,969 with other respiratory tract infection (ORI), and 438,023 controls. Using diagnoses coded according to the German modification of the ICD-10, the associations between prior mental health diagnoses and a PCC diagnosis (primary outcome) or associated symptoms (secondary outcomes) were estimated using multiple Cox proportional hazards regression models. Mental disorders (hazard ratio [HR] 1.36, 95% confidence interval [CI] 1.30–1.42), anxiety (HR 1.14, 95% CI 1.07–1.20), depression (HR 1.25, 95% CI 1.19–1.30) and somatoform disorders (HR 1.30, 95% CI 1.24–1.36) were associated with higher risks for PCC. Mental disorders were associated with the same or even greater risk for a diagnosis of malaise and fatigue in the control cohort (HR 1.71, 95% CI 1.52–1.93) and ORI cohort (HR 1.43, 95% CI 1.20–1.72), than in the COVID-19 cohort (HR 1.43, 95% CI 1.35–1.51). In summary, prior mental comorbidity was associated with an increased risk of PCC and its associated symptoms in all cohorts, not specifically in COVID-19 patients.

## Introduction

More than three years after the start of the global COVID-19 pandemic, a relevant number of people are still suffering from long-term effects, termed "Long-COVID" or "Post-COVID-19 condition" (PCC). Risk factors have been identified in various studies and include female sex, an increased number of symptoms at initial infection and high Body-Mass-Index (BMI)^1^. There is abundant evidence regarding the putative pathomechanisms that play a role in the development of PCC^2^. These include persistent organ damage (especially to the heart, lungs, brain or peripheral nervous system), virus persistence, autoimmune disorder, endothelial or platelet dysfunction and ongoing inflammatory processes^3–5^. Nevertheless, many questions about PCC remain as yet unanswered.

PCC symptoms can be heterogeneous in number, quality and duration^6^. Among the most common PCC symptoms are physical complaints, such as fatigue, memory and concentration disorders, dyspnoea, olfactory and gustatory disturbances^7^. While, in many patients, symptoms improve or resolve, a small subgroup of PCC patients even meet the criteria for the complex and chronic condition Myalgic Encephalomyelitis/Chronic Fatigue Syndrome (ME/CFS)^8^. In addition, some of those affected also suffer from psychological symptoms such as anxiety, depression or sleep disorders^9,10^. A recent longitudinal study from the UK showed that anxiety disorders and depression are more common in COVID-19 patients than in participants without COVID-19^11^. Furthermore, an increased risk of developing mental illness after SARS-CoV-2 infection was shown^12^, and another study found that 64% of patients with unexplained long-term neurological symptoms met the criteria for the presence of somatisation disorder^13^. Beyond this, daily life impairment after SARS-CoV-2 infection was associated with somatic symptom disorder in a local population-based survey^14^. In this context, the question arises whether mental health problems are risk factors for the development of PCC.

PCC is presently considered in the broader context of post-infectious syndromes, as prolonged fatigue can also occur after other acute viral, bacterial or parasitic infectious diseases, such as Epstein-Barr virus, Q-Fever, Dengue infection or Lyme Borreliosis^15–18^. Prolonged postinfectious symptoms are not limited to fatigue. On the one hand, the specific type of infection seems to cause long-term post infectious sequelae. An example is the development of irritable bowel syndrome (IBS) after bacterial gastrointestinal infection with Campylobacter species^19^. There is also evidence that gastrointestinal infections with Giardia lamblia, a parasitic infection, can be associated with the occurrence of Irritable Bowel Syndrome (IBS) and chronic fatigue^20^. On the other hand, in the context of postinfectious fatigue syndromes, psychosocial factors have been identified to play a significant role for the development or perpetuation of symptoms^21^. Fittingly, higher levels of anxiety and depression before an acute infection were also shown to be a predictor for the development of ME/CFS^19^. One of our previous studies demonstrated by means of a routine data analysis that psychological pre-diagnoses and gastrointestinal infections are independent risk factors for the occurrence of both IBS and chronic fatigue syndrome (CFS)^22^. It is therefore of interest to investigate the influence of pre-existing psychological and/or somatisation disorders on PCC.

The analysis of routine data allows to explore the relation of different diagnoses to defined outcomes in large study populations. In the present study, we analysed ambulatory claims data from the Association of Statutory Health Insurance Physicians of Bavaria, which covers all statutorily insured outpatients in Bavaria. The aim was to investigate the association of frequent psychosomatic and/or pre-existing psychiatric diagnoses with a PCC diagnosis in patients infected with SARS-CoV-2, and with the diagnosis of the most common Post-COVID symptoms in two control groups.

## Methods

### Study design and data

A retrospective comparative cohort study was performed using the “Bavarian COVID-19 Cohort” (BCC), a data set derived from the anonymous claims data of all statutory health insurance patients in Bavaria. The BCC data was provided by the National Association of Statutory Health Insurance Physicians of Bavaria (German: Kassenärztliche Vereinigung Bayerns, KVB). Ambulatory care physicians (both GPs and specialists) submit information about treatment, fees and diagnoses for renumeration purposes on a quarterly basis to the KVB. The data covers approximately 85% of the population of Bavaria (11.2 million people with statutory health insurance in 2020)^23^. All diagnoses relevant to a treatment episode are coded according to the German Modification of the International Classification of Diseases (ICD-10-GM). The anonymised claims data provided includes individual information on diagnoses (ICD-10 codes), additional indicators for diagnostic certainty (G: Confirmed, V: Suspicion, Z: Condition after, A: Exclusion), billing quarters, sex, year of birth, participation in a GP-centred care contract (yes/no), physician specialty groups (specialty group ID), care in a nursing home (yes/no), region (rural, urban, city; categories defined according to the regional planning districts of the German Federal Institute for Research on building, urban affairs and spatial development), disbursements (in euros) and codes for items from the Bavarian fee schedule. Data was provided in October, 2022.

Since this is a secondary data use, consent of individuals was not necessary. The handling of the data is based on the guidelines and recommendations of the Good Practice Secondary Data Analysis^24^, as well as all applicable data protection laws and data protection regulations, in particular the Federal Data Protection Act, the Bavarian Data Protection Act and the General Data Protection Regulation (EU) 2016/679. The research project was approved by the Bavarian State Ministry for Health and Care as supervisory authority of the KVB (G35h-A1080-2022/6-5). Corresponding regulations have been defined between the KVB and the MRI in a data supply contract. The study was approved by the ethics committee of the Technical University of Munich (2022-263-S-SR).

### Study cohorts

Data were available for the period January 2015 through June 2022. The BCC data delivered consisted of four groups depending on codes relating to the COVID-19: patients with a confirmed COVID-19 diagnosis, patients with suspected but not confirmed COVID-19, patients with exclusion of COVID-19 and controls with no documented physician contact related to COVID-19. Patients with suspected but not confirmed COVID-19 were excluded from the analysis to guarantee a pure control group.

**Figure 1:**
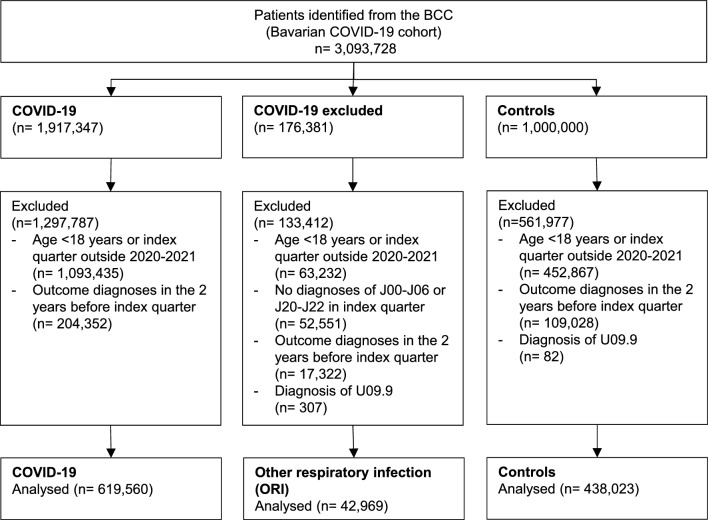
Study Flowchart.

After applying additional inclusion and exclusion criteria, the following three study cohorts were identified (please see Fig. 1 for a study flowchart):

1. *COVID-19* Adult patients with SARS-CoV-2 infection, confirmed by a positive Polymerase Chain Reaction (PCR) test result (U07.1G).
2. *Other respiratory infection (ORI)* Adult patients with the diagnosis “exclusion of COVID-19” (U07.1A), for whom an upper respiratory tract infection (J00-J06) or lower respiratory tract infection (J20-J22) was coded in the same quarter. Influenza and pneumonia (J09-J18) were excluded from the analysis.
3. *Controls* Adult patients without any physician contact related to a confirmed, excluded or suspected COVID-19 infection or other upper or lower respiratory infection. Patients were excluded from this cohort if a diagnosis of PCC was present before the index quarter (assuming that these patients might have had COVID-19 that was documented outside the context of ambulatory care in Bavaria, e.g. a test centre or hospital). Index quarters were defined as outlined below (see section “Observation period”).

Other inclusion criteria were residency in Bavaria at the time of index quarter and quality assurance of the patient ID (consistent data on the date of birth and sex).

Exclusion criteria were age < 18 years and patients with outcome diagnoses coded in the two years before index quarter.

### Observation period

Time was measured in quarters (Q), according to the billing periods. We defined the index quarter (t = 0) as the first quarter with a confirmed COVID-19 diagnosis for the COVID-19 cohort and the first quarter with exclusion of COVID-19 for the ORI cohort, within the period from Q1/2020 to Q4/2021. For the control cohort, an index quarter was allocated by matching the distribution of index quarters of the COVID-19 cohort, ensuring a similar follow-up^23^. The follow-up period was from each index quarter until Q2/2022. The presence of prior mental health diagnoses was assessed in the 5 years prior to the index quarter.

### Outcomes

The primary outcome applicable to the COVID-19 cohort was the diagnosis of a persistent Post-COVID-19 condition, based on the ICD-10 codes U09.9 or U07.4 in at least two quarters following the index quarter. The quarter of the second coding was used to define the time of incidence of the persistent diagnosis. The ICD-10 temporary code U07.4 was introduced in Germany in November 2020. From January 2021 the code for PCC was changed to U09.9 in keeping with the WHO version of the ICD-10.

In order to compare the groups, we defined a set of single symptoms (Table 1) which are among the most common Long-/Post-COVID symptoms (according to the S1-guideline “Long-/Post-COVID”^25^, the WHO-definition of Post-COVID-19 condition^26^ and current literature research^27^), as secondary outcomes. Only persistent symptoms diagnosed in at least two quarters were considered, with the time of incidence given as the second quarter of diagnosis. Only patients with new-onset diagnoses, not coded in the 2 years prior to the index quarter, were included in the analysis.

**Table 1:** Outcome and predictor diagnoses with corresponding ICD-10 Codes according to ICD-10-GM Code Version 2022.

Role	Diagnosis	ICD-10-Code
Outcome	PCC*	U09.9, U07.4
	Malaise and fatigue	R53
	Chronic fatigue syndrome	
	Neurasthenia	F48.0
	Postviral fatigue syndrome/Myalgic encephalomyelitis	G93.3
	Dyspnoea	R06.0
	Other mental disorders due to brain damage and dysfunction and to physical disease	F06
	Mild cognitive disorder	F06.7
	Disturbances of smell and taste	R43
	Cough	R05
	Myalgia	M79.1
	Pulmonary embolism	I26
Predictor	Mental disorders	
	Mood [affective] disorders	F30–F39
	Neurotic, stress-related and somatoform disorders	F40–F48
	Behavioural syndromes associated with physiological disturbances and physical factors)	F50–F59
	Anxiety	
	Other anxiety disorders	F41
	Anxious [avoidant] personality disorder	F60.6
	Separation anxiety disorder of childhood	F93.0
	Phobic anxiety disorder of childhood	F93.1
	Social anxiety disorder of childhood	F93.2
	Depression	
	Depressive episode,	F32
	Recurrent depressive disorder	F33
	Depressive conduct disorder	F92.0
	Somatoform disorders	F45
	Reaction to severe stress, and adjustment disorders	F43
	Diabetes mellitus (Type 1 and 2)	E10–E14
	Asthma	J45
	Obesity	E66
	Hypertension	I10–I15

*PCC Post-Covid-19 condition.

Since the coding of the individual complaints by physicians is not always performed consistently, and in order to differentiate between the various degrees of severity, three different ICD-10 codes (R53, F48.0, G93.3) were included in the analysis for the important symptom complex "fatigue". The diagnosis “Pulmonary embolism” was analysed as a potential complication of COVID-19 infection and served as a plausibility check.

Selected mental health diagnoses, above all anxiety, depression and somatoform disorders each served as independent predictor variable (Table 1). As patients in primary care are often seen in the early stages of their disease, diagnostic uncertainty arises, and there may also be differences in coding practices between physicians^28,29^. Therefore, three less specific superordinate categories of psychological ICD-10 diagnoses were used as predictors in addition to individual diagnoses. The term “Mental disorders” was used as a composite of the superordinate diagnostic categories F30-F59.

We also analysed each of four somatic diagnoses discussed as being risk factors for PCC, as independent predictors: diabetes mellitus, bronchial asthma, obesity and hypertension. This also served as a plausibility check.

### Statistical analysis

The distribution of continuous or categorical data is described by the mean and standard deviation, or absolute and relative frequencies. Statistical significance of differences between cohorts was assessed using t-tests and chi-squared tests. To estimate and test the association of each predictor with primary and secondary outcomes, risk ratios/hazard ratios (HR) with 95% confidence intervals (95% CI) were calculated using Cox proportional hazards regression models with z-tests of the model parameters. Estimates were adjusted for the possible confounders age (years), sex (female/male), number of physician visits 2 years before index quarter, GP-centred care (yes/no), nursing home resident (yes/no), area of residency, treatment costs in the 2 years before index quarter. The proportionality assumption was assessed for each predictor and model by testing and visual inspection of the Schoenfeld residuals in dependence of time. We found no evidence of such a correlation with time, supporting the assumption of proportional risks. Additional risk curves were derived from the models by averaging over the values of the possible confounders, which equals marginal risk estimates. In Bavaria, some patients participate in GP-centered contracts, termed “Hausarztzentrierte Versorgung (HZV)”^30^. GP claims are therefore not available for HZV patients, but GP diagnoses generally are. The BCC data used contains information on the “HZV” status of the patients. In the HZV model, primary care physicians can bill fixed reimbursements once per quarter. The amount varies according to the statutory health insurance. Therefore, treatment costs were corrected by an average of + 60€ per quarter and person participating in a GP-centered care contract. Calculation of treatment costs and the number of physician visits served as a proxy of morbidity. Laboratory cases without physician contact were excluded from this assessment. Incidence rates of outcome diagnoses were calculated in relation to person quarters. Data analysis was performed using R 4.2.2. (The R Foundation for Statistical Computing, Vienna, Austria) with the ICD10gm package for processing ICD-10 metadata^31^. Exploratory and two-sided hypothesis testing was conducted at 5% significance levels.

## Results

Patients with suspected but not confirmed COVID-19 were excluded from the original BCC. The remaining dataset included 3,093,728 patients in total.1,917,347 patients with COVID-19, 176,381 patients with exclusion of COVID-19 and 1,000,000 controls were identified. After applying the inclusion and exclusion criteria, a total of 1,100,552 patients were included in the analysis (Fig. 1). The COVID-19 cohort represented the largest cohort (n = 619,560), followed by controls (n = 438,023) and the ORI cohort (n = 42,969). Distribution of sex, area of residency, ambulatory health care costs and usage were similar between the cohorts. Controls appeared to be about ten years older on average. Table 2 summarises demographic and regional distribution data in the three cohorts prior to the index quarter.

**Table 2:** Baseline characteristics of the cohorts. Descriptive statistics are n (%) or mean ± standard deviation.

Characteristic	COVID-19	ORI*	Control
	n = 619,560	n = 42,969	n = 438,023
Index quarter			
2020/1	27,218 (4.4)	6354 (14.8)	15,051 (3.4)
2020/2	42,273 (6.8)	6943 (16.2)	23,646 (5.4)
2020/3	50,085 (8.1)	8050 (18.7)	30,455 (7.0)
2020/4	127,538 (20.6)	8837 (20.6)	69,082 (15.8)
2021/1	75,470 (12.2)	3370 (7.8)	151,789 (34.7)
2021/2	55,663 (9.0)	1840 (4.3)	29,057 (6.6)
2021/3	37,593 (6.1)	1991 (4.6)	20,179 (4.6)
2021/4	203,720 (32.9)	5584 (13.0)	98,764 (22.5)
Sex			
Female	321,353 (51.9)	22,830 (53.1)	224,558 (51.3)
Age	42.9 (± 16.8)	41.8 (± 16.5)	52.2 (± 19.3)
GP*-centred care	26,417 (4.3)	1647 (3.8)	58,960 (13.5)
Nursing home resident	24,103 (3.9)	954 (2.2)	8043 (1.8)
Area of residency			
City	171,698 (27.7)	14,183 (33.0)	136,728 (31.2)
Region with urbanisation tendencies	118,575 (19.2)	8901 (20.7)	90,801 (20.7)
Rural	328,905 (53.1)	19,862 (46.2)	210,494 (48.1)
Number of physician visits (last two years)	7.4 (± 6.1)	7.9 (± 6.4)	7.1 (± 5.3)
Treatment costs (last 4 quarters, Euro)	552.8 (± 1485.5)	560.4 (± 1089.2)	533.3 (± 986.4)
Follow up time (quarters)	4.5 (± 2.2)	6.1 (± 2.2)	4.8 (± 1.9)

*ORI Other respiratory infection.

*GP General practitioner.

The distribution of patients over the observed index quarters in the COVID-19 cohort corresponded to the course of the infection waves of the COVID-19 pandemic in Bavaria. The quarters 4/2020 and 4/2021 had the highest number of COVID-19 cases in the study population, with n = 127,538 (20.6%) and 203,720 (32.9%), respectively. The lowest number of people tested positive was found in Q1/2020 (n = 27,218, 4.4%). Other respiratory infections with exclusion of COVID-19 occurred twice as frequently in Q1/2020 with n = 6354 (14.8%) compared to Q1/2021 with n = 3370 (7.8%) (Table 3).

**Table 3:** Incidences of primary and secondary outcome diagnoses in the cohorts per 100,000 person years, diagnosed in at least two quarters following the index quarter, during the up to nine quarter follow-up period.

Outcome	Incidences per 100,000 person years		
	COVID-19	ORI*	Controls
PCC*	1400.0	0.0	0.0
Malaise & fatigue	834.4	785.0	221.1
Neurasthenia	474.0	522.3	154.2
Postviral fatigue syndrome	134.8	57.7	22.8
Dyspnoea	664.0	403.9	156.5
Other mental disorders due to brain damage and dysfunction and to physical disease	146.0	91.1	112.8
Mild cognitive disorder	54.9	50.1	62.3
Disturbances of smell and taste	164.9	71.4	15.3
Cough	479.4	473.7	72.3
Myalgia	12.6	13.7	8.6
Pulmonary embolism	93.9	62.3	32.0

*ORI Other respiratory infection.

*PCC Post-COVID-19 condition.

The overall incidence for the primary outcome PCC was 1400/100,000-person years in the COVID-19 cohort. “Malaise and fatigue” and “Neurasthenia” had higher incidences in the COVID-19 and ORI cohort than in the control cohort (Table 3)*.* The diagnosis “Postviral fatigue syndrome” showed a much higher incidence in the COVID-19 cohort, compared to the ORI cohort. Of note is also the increased incidence of “Pulmonary embolism” (93/100,000-person years) compared to the control cohort 32/100,000-person years). An overview of all incidences is shown in Table 3*.*

**Figure 2:**
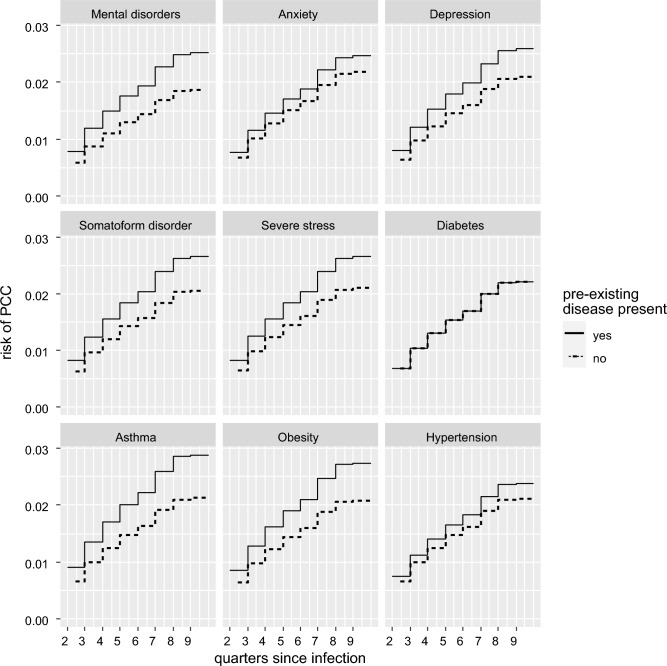
Risk curves for the diagnosis "Post-COVID-19 condition (PCC)” (U09.9, U07.4) in dependence of the predictors “Mental disorders” (F30-F59), “Anxiety” (F41, F60.6, F93.0-F93.2), “Depression” (F32, F33, F92.0), “Somatoform disorders” (F45), “Reaction to severe stress and adjustment disorder” (F43), “Diabetes (Type 1 and 2)” (E10-E14), “Asthma” (J45) and “Obesity” (E66). Estimates are derived from separate models adjusting for potential confounding factors.

Figure 2 shows risk curves for the primary outcome PCC in dependence of all mental health and somatic predictors studied, derived from separate models that corrected for possible confounding (corresponding HRs and p-values are presented in Table 4). The diagnosis of PCC was positively associated in the COVID-19 cohort with the predictors mental disorders (HR 1.36), anxiety (HR 1.14), depression (HR 1.25), somatoform disorders (HR 1.30) and adjustment disorder (HR 1.27). Of the somatic diagnoses included in analysis, bronchial asthma (HR 1.37) and obesity (HR 1.33) showed positive associations, but the risk change was not significant for diabetes mellitus (Table 4)*.*

**Table 4:** HR of the corresponding predictors for the outcome „Post-COVID-19 condition “(U09.9, U07.4) in the COVID-19 cohort. Estimates are derived from separate models for each predictor, adjusted for potential confounding factors.

Predictor variable	Post-COVID-19 condition Hazard Ratio (95% CI)	*p*-value
Mental disorders	1.36 (1.30–1.42)	< 0.001
Anxiety	1.14 (1.07–1.20)	< 0.001
Depression	1.25 (1.19–1.30)	< 0.001
Somatoform disorder	1.30 (1.24–1.36)	< 0.001
Reaction to severe stress, and adjustment disorders	1.27 (1.21–1.33)	< 0.001
Diabetes mellitus (Type 1 and 2)	1.00 (0.94–1.07)	0.918
Asthma	1.37 (1.29–1.45)	< 0.001
Obesity	1.33 (1.26–1.39)	< 0.001
Hypertension	1.13 (1.07–1.19)	< 0.001

**Figure 3:**
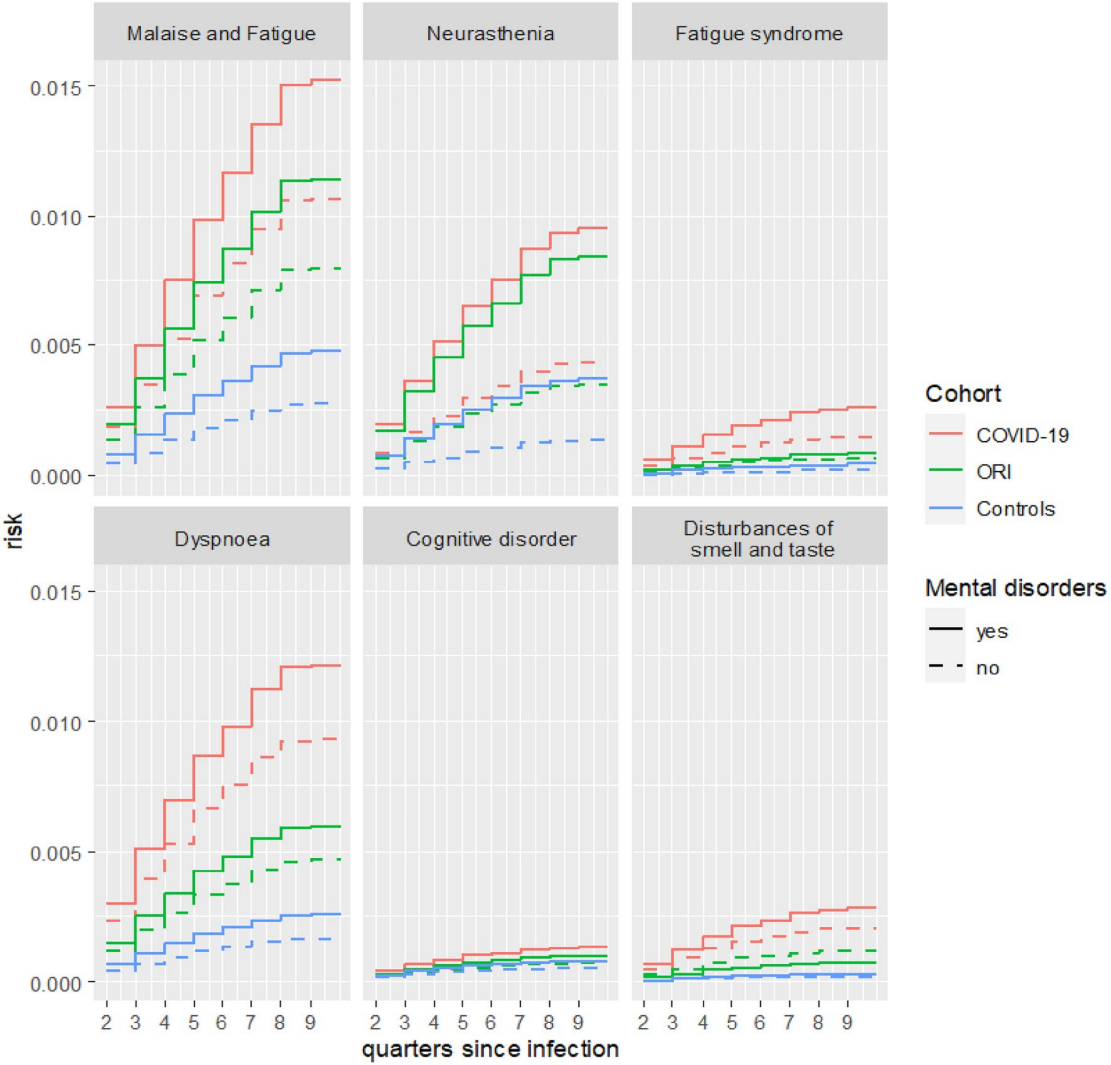
Risk curves for “Malaise and fatigue” (R53), “Neurasthenia” (F48.0), “Postviral fatigue syndrome” (= (G93.3), “Dyspnoea” (R06) and “Mild cognitive disorder” (F06.7) and “Disturbances in smell and taste” (R43) in dependence of "Mental disorders” (F30-F59) in the three comparison cohorts. Estimates are derived from separate models adjusting for potential confounding factors.

Figure 3 shows the risk curves in the three study cohorts (COVID-19, ORI, Controls), with regard to the most important secondary outcome diagnoses “Fatigue” (R53, F48.0, G93.3), “Dyspnoea” (R06.0) and “Mild cognitive disorder” (F06.7) and “Disturbances of smell and taste” (R43) in dependence of the superordinate predictor variable “Mental disorders” (F30-F59). Again, these results were derived from separate models that corrected for possible confounding. The risk increase to develop post-infectious symptoms was highest in the COVID-19 cohort (Fig. 3). However, the presence of “Mental disorders” increased the risk for all cohorts.

Table 5 shows for each of the three cohorts the association, adjusted for possible confounding, of all analysed mental health conditions with the three most frequent PCC associated symptoms "Malaise and fatigue" (R53), "Dyspnoea" (R06) and "Mild cognitive disorder" (F06.7).

**Table 5:** HR of the corresponding predictors for common PCC symptoms (secondary outcomes); “Neurasthenia” (F48) and “Postviral and related fatigue syndrome” (G93.3), representing a more severe course of the symptom fatigue.

	COVID-19	ORI	Controls
	Hazard ratio (95% CI)	Hazard ratio (95% CI)	Hazard ratio (95% CI)
Malaise and fatigue (R53)			
Mental disorder	1.43 (1.35–1.51)	1.43 (1.20–1.72)	1.71 (1.52–1.93)
Anxiety	1.26 (1.18–1.36)	1.53 (1.24–1.89)	1.39 (1.17–1.64)
Depression	1.42 (1.34–1.50)	1.18 (0.98–1.43)	1.59 (1.40–1.81)
Somatoform disorder	1.31 (1.24–1.38)	1.47 (1.23–1.76)	1.54 (1.36–1.75)
Reaction to severe stress, adjustment disorder	1.36 (1.29–1.45)	1.27 (1.05–1.53)	1.43 (1.25–1.65)
Neurasthenia (F48)			
Mental disorder	2.21 (2.04–2.39)	2.42 (1.89–3.11)	2.77 (2.37–3.23)
Anxiety	1.57 (1.44–1.72)	1.37 (1.05–1.78)	2.18 (1.84–2.59)
Depression	1.91 (1.77–2.05)	1.62 (1.30–2.01)	2.15 (1.86–2.49)
Somatoform disorder	1.64 (1.53–1.77)	1.69 (1.37–2.10)	2.02 (1.75–2.33)
Reaction to severe stress, adjustment disorder	1.83 (1.70–1.97)	1.89 (1.52–2.34)	2.34 (2.02–2.72)
Postviral and related fatigue syndrome (G93.3)			
Mental disorder	1.72 (1.49–1.98)	2.42 (1.89–3.11)	1.96 (1.34–2.86)
Anxiety	1.25 (1.05–1.48)	1.28 (0.59–2.80)	1.48 (0.90–2.41)
Depression	1.79 (1.56–2.05)	1.51 (0.79–2.90)	2.06 (1.42–2.97)
Somatoform disorder	1.44 (1.26–1.65)	1.78 (0.94–3.37)	1.88 (1.29–2.72)
Reaction to severe stress, adjustment disorder	1.59 (1.39–1.83)	1.75 (0.91–3.35)	2.11 (1.42–3.13)
Dyspnoea (R06)			
Mental disorder	1.31 (1.23–1.39)	1.28 (1.00–1.65)	1.55 (1.35–1.79)
Anxiety	1.14 (1.06–1.24)	1.37 (1.02–1.84)	1.21 (0.99–1.49)
Depression	1.22 (1.14–1.30)	1.21 (0.94–1.56)	1.56 (1.34–1.80)
Somatoform disorder	1.32 (1.24–1.40)	1.18 (0.92–1.52)	1.67 (1.44–1.93)
Reaction to severe stress, adjustment disorder	1.23 (1.15–1.31)	1.07 (0.82–1.40)	1.24 (1.04–1.48)
Mild cognitive disorder (F06.7)			
Mental disorder	1.87 (1.49–2.35)	1.40 (0.67–2.96)	1.46 (1.17–1.83)
Anxiety	1.81 (1.42–2.31)	1.87 (0.87–4.04)	1.37 (1.00–1.87)
Depression	2.08 (1.69–2.56)	2.03 (1.02–4.03)	1.52 (1.21–1.91)
Somatoform disorder	1.50 (1.22–1.85)	1.10 (0.54–2.25)	1.35 (1.06–1.71)
Reaction to severe stress, adjustment disorder	1.67 (1.33–2.09)	1.05 (0.47–2.33)	1.68 (1.28–2.20)
Disturbances of smell and taste (R43)			
Mental disorder	1.39 (1.22–1.56)	0.62 (0.35–1.10)	1.47 (0.95–2.31)
Anxiety	1.27 (1.08–1.49)	0.96 (0.43–2.15)	1.08 (0.53–2.16)
Depression	1.24 (1.09–1.42)	0.83 (0.42–1.64)	1.45 (0.89–2.36)
Somatoform disorder	1.39 (1.23–1.58)	1.83 (0.42–1.64)	1.15 (0.69–1.91)
Reaction to severe stress, adjustment disorder	1.44 (1.26–1.64)	0.78 (0.39–1.58)	1.62 (0.97–2.72)

Estimates are derived from separate models for each predictor, adjusted for potential confounding factors.

*ORI* Other respiratory infection.

Overall it can be noted that the psychological predictors were associated with similar risk increases for the diagnoses mentioned above, across all cohorts. For certain diagnoses, the risk increase was even higher in the control cohort, than in the COVID-19 and ORI cohort. The largest increase in risk occurred for the outcome “Neurasthenia” in presence of “Mental disorder”. An overview of Hazard ratios for all predictors and outcomes studied is provided in the Supplementary Table S1.

## Discussion

In summary, COVID-19 patients were at increased risk of prolonged postinfectious symptoms in the comparison of cohorts. Pre-existing mental health diagnoses were associated with an increased risk of PCC. However, the increase in risk due to pre-existing mental health diagnoses, was the same or even higher in control cohorts without SARS-CoV-2 infection for individual PCC associated symptoms such as cognitive impairment, dyspnoea, and the symptom complex “fatigue”. Therefore, the association between pre-existing mental health problems and post-infectious symptoms does not seem to be specific to Post-COVID-19 patients.

A possible explanation for the increase in risk for the examined symptom diagnoses in the cohort of controls is the assumption that patients with pre-existing mental health diagnoses more frequently consult general practitioners. This increases the probability for this group to be diagnosed with one of the examined somatic complaints. An increased utilisation of medical services is known especially in the presence of a somatic symptom disorder^29^. However, the fact that no specific association of mental pre-diagnoses and PCC symptoms could be found in the cohort comparison underlines the importance of further research into the underlying pathomechanisms of PCC. As an explanation for prolonged cognitive symptoms, for example, correlates could be found in MRI studies^32^. Further immunological or molecular biological mechanisms^33,34^ in connection with PCC are in the process of being more fully understood. As the field of post-acute infectious syndromes has a lot of research gaps, the increased attention due to PCC could contribute to deepening the immunological understanding of these conditions^20^. On the other hand, our analysis has shown that psychological (co-)morbidity plays an important role in the development of post-infectious symptoms, which should also be considered in future research. Negative expectations in the course of the infection due to pre-existing psychological impairments could be influencing factors for the maintenance of symptoms. Fittingly, aspects of a somatic symptom disorder, i.e. a combination of suffering from physical complaints, regardless of their origin, with psychological behavioural features of an excessive preoccupation with one's own physical symptoms and accompanied by increased psychological distress^35^, should be considered in patient counselling^14^. In this context, it should also be noted that both the quarantine measures^36^ and the experience of the pandemic itself^37^ led to increased psychological vulnerability. These could be confounding variables that cannot be controlled. Regardless, our results show that knowledge of pre-existing mental (co-)morbidity could help identify at-risk patients to ensure optimal supportive care after infectious disease. Qualitative studies may be needed to gain a more comprehensive understanding of patients' experiences and the impact of mental health diagnoses on living with PCC.

With regard to the results of the analysed somatic predictors, diabetes mellitus did not show an increased risk for PCC. A similar conclusion is drawn from the case–control study by Fernandez-de-las-Penas et al^38^. As there is evidence that oral antidiabetic treatment, such as metformin, offers potentially beneficial effects in the course of mild to moderate COVID-19 infections, this could be a possible explanation for the observed results^39^. Nevertheless, the results in our study were not significant. Asthma and obesity showed significantly increased HRs for the development of PCC, which is plausible as both diagnoses are being discussed as risk factors for PCC^40–42^. The risk increase for a diagnosis of Post-Covid-19 condition with pre-existing hypertension is plausible, as arterial hypertension is recognised as a risk factor for a more severe course of COVID-19^43^. Remarkable, irrespective of the mental comorbidities, is the increased incidence of the diagnosis “pulmonary embolism” in the COVID-19 cohort. Thromboembolic complications are a known risk factor of COVID-19 disease^44^.

The increase in the relative risk of developing symptoms due to pre-existing mental health diagnoses was substantial for the diagnoses studied, but the absolute risk was comparatively low. Regarding the incidences in the follow-up period, the difference for the outcome diagnosis “Malaise and fatigue” between the COVID-19 (834/100,000 person-years) and the ORI (785/100,00 person-years) cohort does not seem to be high. Thus, the impact of SARS-CoV-2 infection on prolonged fatigue appears to be limited compared to other respiratory infections. But considering the high incidences of SARS-CoV-2 infections throughout the pandemic, the impact on the overall population is substantial. As many infections were not documented by PCR, the real prevalence of COVID-19 is still unclear. But if we calculate our incidence for “Malaise & fatigue” with the total number of inhabitants of Bavaria (approximately 13 Million people), we can estimate that the incidence would be about 108,420 additional cases in one year. And regarding the more severe form “Postviral and related fatigue syndrome”, the difference was higher in the COVID-19 cohort (134/100,000-person years), which would result in approximately 17,420 severely affected cases in Bavaria within one year. This can potentially explain the controversy, why PCC does not appear as something frequent for the individual GP on the one hand, but is also discussed as having a huge impact on health services on the other hand.

### Strengths and limitations

Our study shows the strengths and limitations typical of routinely collected data use. This must be considered while interpreting our results. A strength is the high number of cases. Regarding external validity, results from a primary care population sample allow more generalisability than studies conducted in specialised and/or hospital settings. However, we cannot completely rule out the possibility of a selection bias, as the data source approximately covers only 85% of the population of Bavaria, and because of healthcare-seeking behaviour that may be reflected in the data. We had access to data from the last 5 years for the retrograde analysis of preliminary diagnoses, therefore a differentiation between pre-existing and new onset conditions was possible. The definition of a diagnosis is based on the ICD-codes defined by GPs and/or specialists. This allows a more objective analysis than self-reporting questionnaires. The individual coding habits of the physicians may however vary and information on clinical data is not available. We tried to minimise accidental diagnoses by only including secured diagnoses in the analysis, and outcomes had to be coded in a minimum of two quarters. We assume that a certain number of PCC patients might be missing, as the first PCC specific ICD-10 code U07.4 was introduced in Germany in November 2020. First PCC cases most likely presented before this date. However, the overall number of SARS-CoV-2 infections from March 2020–October 2020 was significantly lower than in the following quarters. We excluded influenza from the “other respiratory infection” analysis, as due to the public health control measures the incidence of influenza was extremely low in Bavaria, respectively in Germany, than in previous years. Although claims data were available until Q2/2022, we limited the inclusion of index dates from the beginning of the pandemic until the start of the Omicron wave in December 2021. At this point, the number of PCR tests performed no longer correlated with the actual number of COVID-19 patients, as due to mild symptoms and eased pandemic restrictions PCR testing was no longer performed in every affected individual. The symptoms selected as secondary outcomes do not represent the full spectrum of PCC, but were limited to the most common symptoms according to current literature research. Considering the high number of symptoms that can potentially be experienced by PCC patients, this seemed to be a reasonable and pragmatic approach. It should be noted that for the very common and important main PCC symptoms “fatigue” or “brain fog”, there is no specific code in the ICD-10 system. Thus, different codes were chosen for the analysis, that resemble the symptoms most closely. In this respect it is important to note that the corresponding results need to be interpreted in the context of the codes used and should not be extrapolated to definitions potentially including other codes. Effect estimation was conducted separately for each of the investigated predictors adjusting for possible confounding. The latter included the number of physician visits and costs as a proxy of morbidity. However, further multivariable analyses and exploration of effect modification and interaction in dependence of comorbidities, for example concerning the somatic diagnosis of diabetes, have not been performed and have to remain subject of future research. Another limitation in the same line of reasoning is that it was not possible in a reliable way to consider the treatment of the diseases studied in the analyses, such as the type of medication and adherence to it. Finally, we cannot exclude that our findings are influenced by regional healthcare practices and patient demographics in Bavaria, which may limit their generalizability.

## Conclusion

To summarise our study findings, pre-existing psychological and psychosomatic diagnoses are associated with an increased risk for the physical complaints studied, but this is not specific to COVID-19 patients, who nevertheless showed the highest burden of disease. On the one hand, psychological and psychosomatic complaints need to be adequately addressed in some PCC patients. On the other hand, our results can be interpreted to imply that prior mental health diagnoses play a similar role in the explanation of disease burden in PCC patients, as in other diseases. These relations indicate that patients should be treated in terms of a comprehensive bio-psycho-social understanding, taking into account any pre-existing mental health conditions to ensure optimal supportive treatment.

### Supplementary Information

Supplementary Information.

## Data Availability

The underlying data is not distributed for data protection reasons. Access may be granted upon reasoned request to the corresponding author or to the Bavarian Association of Statutory Health Insurance Physicians.
