# Supplementary material for: Impact of pre-existing mental health diagnoses on development of post-COVID and related symptoms: a claims data-based cohort study
Source: Sci Rep. 2024 Jan 29;14:2408. doi: 10.1038/s41598-024-52656-6 (PMC10824734; doi:10.1038/s41598-024-52656-6)
Supplement: Supplementary file 1 — Supplementary Information. [file 41598_2024_52656_MOESM1_ESM.pdf]

Supplementary Information

Supplementary Table S1 (A-C) - *All Hazard ratios (HR) with 95% confidence interval (CI) of secondary outcomes in dependence of predictor variables in the three study cohorts, sorted by the main clusters of symptoms of Post-COVID-19 condition*

A) Cluster of symptoms: "Fatigue"

		**Malaise and fatigue** HR (95% CI)	**Neurasthenia** HR (95% CI)	**Postviral fatigue syndrome** HR (95% CI)
**Mental & behavioural disorder**	Covid-19	1.43 (1.35, 1.51)	2.21 (2.04, 2.39)	1.72 (1.49, 1.98)
	Controls	1.71 (1.52, 1.93)	2.77 (2.38, 3.23)	1.96 (1.34, 2.86)
	ORI	1.43 (1.2, 1.72)	2.42 (1.89, 3.11)	1.27 (0.65, 2.49)
**Anxiety**	Covid-19	1.26 (1.18, 1.36)	1.57 (1.44, 1.72)	1.25 (1.05, 1.48)
	Controls	1.39 (1.17, 1.64)	2.18 (1.84, 2.59)	1.48 (0.9, 2.41)
	ORI	1.53 (1.24, 1.89)	1.37 (1.05, 1.78)	1.28 (0.59, 2.8)
**Depression**	Covid-19	1.42 (1.34, 1.5)	1.91 (1.77, 2.05)	1.79 (1.56, 2.05)
	Controls	1.59 (1.4, 1.81)	2.15 (1.87, 2.49)	2.06 (1.42, 2.97)
	ORI	1.18 (0.98, 1.43)	1.62 (1.3, 2.01)	1.51 (0.79, 2.9)
**Somatoform disorder**	Covid-19	1.31 (1.24, 1.38)	1.64 (1.53, 1.77)	1.44 (1.26, 1.65)
	Controls	1.54 (1.36, 1.75)	2.02 (1.75, 2.33)	1.88 (1.29, 2.72)
	ORI	1.47 (1.23, 1.76)	1.7 (1.37, 2.1)	1.78 (0.94, 3.37)
**Adjustment disorder**	Covid-19	1.36 (1.29, 1.45)	1.83 (1.71, 1.97)	1.6 (1.39, 1.83)
	Controls	1.43 (1.25, 1.65)	2.34 (2.02, 2.72)	2.11 (1.42, 3.13)
	ORI	1.27 (1.05, 1.53)	1.89 (1.52, 2.34)	1.75 (0.91, 3.35)
**Diabetes**	Covid-19	0.90 (0.82, 1.00)	0.76 (0.66, 0.87)	0.76 (0.6, 0.96)
	Controls	1.04 (0.88, 1.23)	0.57 (0.45, 0.73)	0.85 (0.52, 1.39)
	ORI	0.65 (0.45, 0.95)	0.78 (0.52, 1.18)	0.9 (0.32, 2.55)
**Asthma**	Covid-19	1.05 (0.97, 1.13)	1.13 (1.02, 1.25)	1.38 (1.16, 1.64)
	Controls	1.03 (0.85, 1.26)	1.3 (1.05, 1.61)	1.71 (1.03, 2.82)
	ORI	0.89 (0.69, 1.16)	0.85 (0.62, 1.18)	0.48 (0.15, 1.56)
**Obesity**	Covid-19	0.98 (0.91, 1.05)	1.11 (1.02, 1.21)	1.18 (1.01, 1.38)
	Controls	0.99 (0.85, 1.16)	0.94 (0.78, 1.14)	1.13 (0.72, 1.77)
	ORI	0.81 (0.63, 1.05)	1.13 (0.86, 1.49)	1.39 (0.66, 2.95)
**Hypertension**	Covid-19	0.93 (0.86, 0.99)	0.95 (0.87, 1.04)	1.03 (0.87, 1.21)
	Controls	1.07 (0.95-1.22)	0.82 (0.71, 0.96)	0.88 (0.61, 1.29)
	ORI	0.85 (0.69, 1.05)	1.04 (0.81, 1.32)	1.77 (0.93, 3.38)

B) Cluster of symptoms: „Respiratory“

		**Dyspnoea**<br>HR (95% CI)	**Cough**<br>HR (95% CI)	**Pulmonary embolism**<br>HR (95% CI)
**Mental & behavioural disorder**	Covid-19	1.31 (1.23, 1.39)	1.35 (1.26, 1.45)	1.08 (0.92, 1.27)
	Controls	1.55 (1.35, 1.79)	1.46 (1.19, 1.79)	0.99 (0.73, 1.34)
	ORI	1.28 (1, 1.65)	1.26 (1, 1.58)	1.36 (0.71, 2.6)
**Anxiety**	Covid-19	1.14 (1.06, 1.24)	1.26 (1.14, 1.39)	0.98 (0.78, 1.24)
	Controls	1.21 (0.99, 1.49)	1.18 (0.86, 1.61)	0.89 (0.52, 1.51)
	ORI	1.37 (1.02, 1.84)	1.09 (0.81, 1.48)	1.83 (0.89, 3.73)
**Depression**	Covid-19	1.22 (1.14, 1.3)	1.32 (1.22, 1.43)	1.08 (0.91, 1.29)
	Controls	1.56 (1.34, 1.8)	1.38 (1.1, 1.73)	1.2 (0.86, 1.68)
	ORI	1.21 (0.94, 1.56)	1.08 (0.84, 1.38)	1.3 (0.69, 2.46)
**Somatoform disorder**	Covid-19	1.32 (1.24, 1.4)	1.31 (1.22, 1.41)	1.09 (0.91, 1.29)
	Controls	1.67 (1.44, 1.93)	1.29 (1.03, 1.63)	1.41 (1.01, 1.97)
	ORI	1.18 (0.92, 1.52)	1.28 (1.01, 1.61)	1.56 (0.84, 2.91)
**Adjustment disorder**	Covid-19	1.23 (1.15, 1.31)	1.25 (1.15, 1.35)	1.15 (0.95, 1.39)
	Controls	1.24 (1.04, 1.48)	1.37 (1.07, 1.76)	1.07 (0.69, 1.65)
	ORI	1.07 (0.82, 1.4)	1.17 (0.92, 1.5)	1.25 (0.63, 2.5)
**Diabetes**	Covid-19	0.97 (0.89, 1.06)	0.9 (0.79, 1.03)	0.9 (0.73, 1.12)
	Controls	1.4 (1.2, 1.64)	1.14 (0.86, 1.51)	0.73 (0.5, 1.06)
	ORI	1.05 (0.74, 1.48)	1.16 (0.79, 1.69)	0.62 (0.24, 1.58)
**Asthma**	Covid-19	1.46 (1.35, 1.58)	1.31 (1.19, 1.44)	1.43 (1.16, 1.77)
	Controls	2.02 (1.69, 2.43)	2.34 (1.81, 3.03)	1.89 (1.24, 2.87)
	ORI	0.99 (0.7, 1.39)	0.89 (0.64, 1.24)	1.23 (0.55, 2.79)
**Obesity**	Covid-19	1.44 (1.34, 1.53)	1.24 (1.14, 1.35)	1.62 (1.36, 1.91)
	Controls	1.89 (1.63, 2.2)	1.15 (0.88, 1.5)	1.92 (1.38, 2.66)
	ORI	1.36 (1.03, 1.81)	1.01 (0.75, 1.38)	1.34 (0.66, 2.74)
**Hypertension**	Covid-19	1.12 (1.04,1.20)	1.13 (1.04, 1.24)	1.20 (1.00, 1.45)
	Controls	2.11 (1.81, 2.46)	1.57 (1.27, 1.94)	0.89 (0.65, 1.23)
	ORI	1.51 (1.18, 1.93)	1.10 (0.85, 1.43)	2.63 (1.23, 4.54)

C) *Cluster of symptoms „Cognitive disorder / Neurological"*

		**Mental disorder due to brain dysfunction and to physical disease** HR (95% CI)	**Mild cognitive disorder** HR (95% CI)	**Myalgia** HR (95% CI)	**Disturbances of smell and taste** HR (95% CI)
**Mental & behavioural disorder**	Covid-19	1.65 (1.43, 1.89)	1.87 (1.49, 2.35)	1.11 (0.72, 1.71)	1.38 (1.22, 1.56)
	Controls	1.64 (1.38, 1.94)	1.46 (1.17, 1.83)	1.98 (1.06, 3.67)	1.49 (0.95, 2.31)
	ORI	1.21 (0.71, 2.08)	1.4 (0.67, 2.96)	0.38 (0.09, 1.51)	0.62 (0.35, 1.1)
**Anxiety**	Covid-19	1.48 (1.26, 1.73)	1.81 (1.42, 2.31)	1.14 (0.63, 2.08)	1.27 (1.08, 1.49)
	Controls	1.6 (1.29, 2)	1.37 (1, 1.87)	1.28 (0.54, 3.04)	1.08 (0.54, 2.16)
	ORI	1.13 (0.59, 2.18)	1.87 (0.87, 4.04)	0 (0, Inf)	0.96 (0.43, 2.15)
**Depression**	Covid-19	1.65 (1.46, 1.88)	2.08 (1.69, 2.56)	1.1 (0.68, 1.79)	1.24 (1.09, 1.42)
	Controls	1.63 (1.38, 1.93)	1.52 (1.21, 1.91)	1.36 (0.71, 2.61)	1.45 (0.89, 2.36)
	ORI	1.69 (1.02, 2.81)	2.03 (1.02, 4.03)	0 (0, Inf)	0.83 (0.42, 1.64)
**Somatoform disorder**	Covid-19	1.36 (1.19, 1.55)	1.5 (1.22, 1.85)	1.58 (1.02, 2.47)	1.39 (1.23, 1.58)
	Controls	1.38 (1.15, 1.64)	1.35 (1.06, 1.71)	1.51 (0.8, 2.87)	1.15 (0.69, 1.91)
	ORI	0.95 (0.55, 1.64)	1.1 (0.54, 2.25)	1.15 (0.29, 4.61)	0.98 (0.52, 1.83)
**Adjustment disorder**	Covid-19	1.4 (1.21, 1.62)	1.67 (1.33, 2.09)	0.82 (0.48, 1.41)	1.44 (1.26, 1.64)
	Controls	1.75 (1.44, 2.14)	1.68 (1.28, 2.2)	1.11 (0.51, 2.39)	1.62 (0.97, 2.72)
	ORI	0.98 (0.53, 1.81)	1.05 (0.47, 2.33)	0 (0, Inf)	0.78 (0.39, 1.58)
**Diabetes**	Covid-19	1.17 (1.02, 1.34)	1.09 (0.86, 1.39)	0.89 (0.43, 1.83)	0.62 (0.47, 0.8)
	Controls	1.25 (1.05, 1.48)	1.14 (0.9, 1.45)	1 (0.45, 2.21)	0.97 (0.51, 1.84)
	ORI	1.44 (0.83, 2.48)	1.32 (0.61, 2.85)	0.98 (0.12, 7.93)	0.74 (0.23, 2.39)
**Asthma**	Covid-19	1.1 (0.91, 1.34)	1.44 (1.1, 1.89)	0.72 (0.35, 1.5)	0.96 (0.8, 1.15)
	Controls	0.98 (0.74, 1.31)	1.06 (0.73, 1.52)	3.58 (1.85, 6.95)	1.95 (1.08, 3.54)
	ORI	1.19 (0.61, 2.36)	0.97 (0.37, 2.51)	1.71 (0.35, 8.25)	1.87 (0.95, 3.68)
**Obesity**	Covid-19	1.14 (0.98, 1.32)	1.13 (0.9, 1.43)	1.34 (0.82, 2.21)	0.98 (0.84, 1.14)
	Controls	1.23 (1.01, 1.49)	1.37 (1.07, 1.76)	0.83 (0.37, 1.88)	1.19 (0.68, 2.09)
	ORI	1.4 (0.79, 2.48)	1.66 (0.79, 3.48)	0.58 (0.07, 4.66)	1.6 (0.81, 3.14)
**Hypertension**	Covid-19	1.37 (1.17, 1.60)	1.23 (0.97, 1.57)	1.05 (0.63, 1.74)	0.95 (0.81, 1.12)
	Controls	1.37 (1.12, 1.67)	1.42 (1.09, 1.86)	0.77 (0.40, 1.49)	1,22 (0.78, 1.93)
	ORI	1.08 (0.65, 1.82)	1.33 (0.66, 2.69)	0.27 (0.03, 2.22)	0.73 (0.35, 1.52)
